# Evaluation of Spino-Pelvic Parameters in Asian Patients Undergoing Total Hip Arthroplasty

**DOI:** 10.7759/cureus.101047

**Published:** 2026-01-07

**Authors:** Adarsh Annapareddy, Tarun Jayakumar, Praharsha Mulpur, Vemaganti Badri Narayana Prasad, Mahesh Kulkarni, Arun Kannan, Ravikumar Mukartihal, Sharan S Patil, Rohit Rakesh Luthra, Ashish Singh, Aditya Seth, Mujtaba Ansari, A. V. Gurava Reddy

**Affiliations:** 1 Orthopaedics, Sunshine Bone and Joint Institute, KIMS-Sunshine Hospitals, Hyderabad, IND; 2 Orthopaedics, Deenanath Mangeshkar Hospital, Pune, IND; 3 Orthopaedic Surgery, Apollo Hospitals, Chennai, IND; 4 Orthopaedics, Sparsh Hospital, Bangalore, IND; 5 Orthopaedics, Arcus Hospital, Pune, IND; 6 Orthopaedics, Anup Institute of Orthopaedics and Rehabilitation, Patna, IND

**Keywords:** hip-spine relationship, pelvic incidence, sacral slope, sagittal balance, spino-pelvic parameters, total hip arthroplasty

## Abstract

Introduction: Emerging research emphasizes the critical relationship between the hip and spine affecting implant positioning in total hip arthroplasty (THA). While spino-pelvic parameters and mobility patterns have been well described in Western populations, corresponding data from Asian patients undergoing THA remain limited. The primary objective of this study was to evaluate preoperative spino-pelvic parameters in Asian patients undergoing THA. The secondary objectives were to classify these patients using the hip-spine classification system and to compare the observed distribution with existing literature.

Methods: A multicenter, retrospective observational study was conducted across six high-volume centers from June 2023 to December 2024. Patients aged ≥18 years undergoing primary THA with available flexed-seated and standing lateral lumbosacral spine radiographs were included. Radiographic parameters were independently measured by two observers, patients were categorized according to the hip-spine classification, and statistical analyses were performed.

Results: A total of 836 patients (mean age: 46.35±14.4 years; 72% male) were eligible for final evaluation. Avascular necrosis of the femoral head was the predominant indication (87.1%). Radiographic measurements showed excellent reproducibility (intraclass correlation coefficient (ICC)=0.82). Distribution according to hip-spine classification was: 1A (78.9%, N=660), 1B (9.6%, N=80), 2A (8%, N=67), and 2B (3.5%, N=29). Mean pelvic incidence, lumbar lordosis, standing sacral slope, and sitting sacral slope were 48.78°±10°, 53.88°±9.95°, 38.82°±8.85°, and 14.61°±11.14°, respectively.

Conclusion: This study provides a large, multicenter descriptive analysis of spino-pelvic parameters in an Asian THA population and demonstrates a predominance of normal spino-pelvic alignment and mobility patterns when classified using the hip-spine classification system. The observed distribution differs from that reported in Western cohorts, likely reflecting regional and etiological differences. These findings offer a foundational understanding of spino-pelvic characteristics in Asian patients undergoing THA and highlight the need for future longitudinal studies correlating spino-pelvic classification with postoperative clinical outcomes.

## Introduction

Total hip arthroplasty (THA) is a well-established and reliable surgical intervention for managing end-stage arthritis of the hip joint. With increasing life expectancy, there is a growing elderly population in many countries, leading to a higher prevalence of degenerative conditions affecting the hip and spine [1,2]. Recognized as the operation of the century, THA consistently demonstrates excellent outcomes and a high rate of functional recovery across all age groups [3].

A significant complication encountered by arthroplasty surgeons is dislocation following THA, which can have serious consequences [4,5]. Component malpositioning is a critical technical factor contributing to impingement and subsequent dislocation. Substantial research has been dedicated to identifying optimal component positioning, with intraoperative guidelines developed to achieve these parameters. Lewinnek's "safe zone" for acetabular component positioning is widely cited for its potential to reduce dislocation risk [6]. However, recent studies have highlighted limitations in this concept, reporting high dislocation rates even when components are positioned within the proposed safe zone [7,8].

The hip-spine relationship has garnered significant attention, particularly following Offierski and McNab's introduction of "hip-spine syndrome," which illustrates that degenerative spine disease can lead to hip joint degeneration and studies showed higher hip degeneration in patients who underwent spinal fusion surgeries in the past [9,10]. Vigdorchik et al. have emphasized the notable changes in the functional positioning of the acetabular component with different postures, such as transitioning from supine to sitting or standing positions [11].

The advent and increased adoption of robotics in hip arthroplasty have enabled individualized functional cup positioning and virtual intraoperative impingement assessment, aiming to reduce dislocation risk after THA [12-14]. However, the use of technology without a proper understanding of the spino-pelvic parameters of our patients is sub-optimal. Vigdorchik et al. introduced a hip-spine classification for patients undergoing THA [15]. While some studies have reported spino-pelvic radiological parameters in healthy, non-arthritic Indian patients, there is a lack of published data on these parameters and spino-pelvic mobility classification in Asian patients undergoing THA [16-18].

The aim of this study was to evaluate the spino-pelvic relationship in Asian patients undergoing total hip arthroplasty. The secondary objectives of the study were to report the spino-pelvic parameters of Asian patients using the hip-spine classification and compare them with other populations.

## Materials and methods

This was a multicenter retrospective observational study conducted across six high-volume centers performing primary total hip arthroplasty between June 2023 and December 2024. After obtaining Institutional Ethics Committee approval (SIEC/2023/532), anonymized demographic data were collected from the electronic health records (EHR), institutional arthroplasty database and radiological images were obtained from the medical picture archives (PACS).

**Figure 1:**
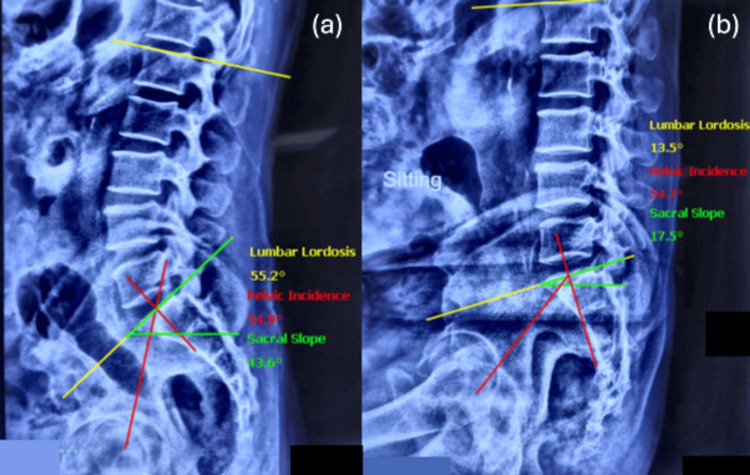
Spino-pelvic parameters--lumbar lordosis, pelvic incidence, and sacral slope--were measured pre-operatively in standing (a) and flexed-seated (b) positions.

All patients aged 18 years or older undergoing primary THA with available sitting and standing lateral lumbosacral spine radiographs consenting for the use of their radiological data were included in the study. Patients undergoing revision THA, previous history of spine surgeries, and patients below the age of 18 years were excluded from the study. Preoperative radiological evaluation included plain radiographs of the pelvis with both hips in antero-posterior view, and a lateral view x-ray of the lumbosacral spine in sitting and standing positions. All measurements were performed on the institutional Picture Archiving and Communication System (PACS, Siemens Healthineers, Erlangen, Germany) (Figure 1).

Radiographic analysis

All radiographic parameters were measured by two separate observers independently to address inter-observer and intra-observer variability, and the average of the two sets of measurements was used for subsequent analysis.

To minimize inter-center variability, a standardized radiographic acquisition protocol was implemented across all participating centers. Standing lateral lumbosacral radiographs were obtained with patients in an upright, relaxed posture, while seated radiographs were acquired in a flexed-seated position with the hips and knees flexed to approximately 90°. Patients were instructed to maintain a neutral trunk position without external support. Radiographs were centered on the lumbosacral junction and acquired at a consistent source-to-image distance, ensuring uniform assessment of spino-pelvic alignment and mobility.

Spino-pelvic parameters were measured using standardized anatomical landmarks. Pelvic incidence was defined as the angle between a line perpendicular to the midpoint of the sacral endplate and a line connecting this point to the center of the femoral head. Lumbar lordosis was measured as the Cobb angle between the superior endplate of L1 and the superior endplate of S1. Sacral slope was defined as the angle between the superior sacral endplate and the horizontal plane, measured in both standing and flexed-seated positions. Spino-pelvic mobility was calculated as the change in sacral slope between standing and seated radiographs.

Classification of the hip-spine relationship

All patients were classified using the hip-spine classification system proposed by Vigdorchik et al. [15] into four groups: Group 1 with normal spinal alignment; Group 2 with a flatback deformity--Group 2A with normal spinal mobility, and Group 2B with a stiff spine. Flatback deformity was defined as a pelvic incidence minus lumbar lordosis of more than 10°, and spinal stiffness was defined as less than a 10° change in sacral slope from standing to seated.

Statistical analysis

Continuous variables were depicted as means with accompanying standard deviations, while categorical variables were articulated using frequencies and proportions. Inter-observer and intra-observer reliability for all continuous spino-pelvic radiographic measurements was assessed using the intraclass correlation coefficient (ICC). ICC values were interpreted according to established thresholds, with values >0.75 indicating good reliability. A p-value of less than 0.05 was considered statistically significant. The statistical analysis was performed utilizing Statistical Package for Social Sciences (SPSS) version 24 (IBM Corp., Armonk, NY).

## Results

The final analysis included 836 consecutive patients who underwent primary total hip arthroplasty. The majority of the patient population was males (N=602, 72%), with a mean age of 46.35 (SD=14.4) years. Out of the 836 cases, 74 (8.8%) were bilateral, 402 (48.1%) were on the left side, and 360 (43.1%) were operated on the right side. The majority of the cases were caused by avascular necrosis (AVN) of the femur head (87.1%, N=728), while post-traumatic arthritis, neck of femur fracture, and dysplastic hips were the other common causes, as shown in Table 1.

**Table 1:** Demographics of the patient population.

Parameter	Mean	SD
Age (years)	46.35	14.4
BMI (kg/m^2^)	28.14	10.2
	Number	Percentage
Gender, male	602	72
Side		
Right	360	43.1
Left	402	48.1
Bilateral	74	8.8
Etiology		
Avascular necrosis	728	87.1
Post-traumatic arthritis	68	8.1
Dysplastic hip	25	3
Femur neck fracture	15	1.8

Excellent reproducibility was exhibited by the ICC value of 0.82 (95% CI: 0.86-0.90) for internal consistency and observer reliability in measuring the spino-pelvic parameters.

**Figure 2:**
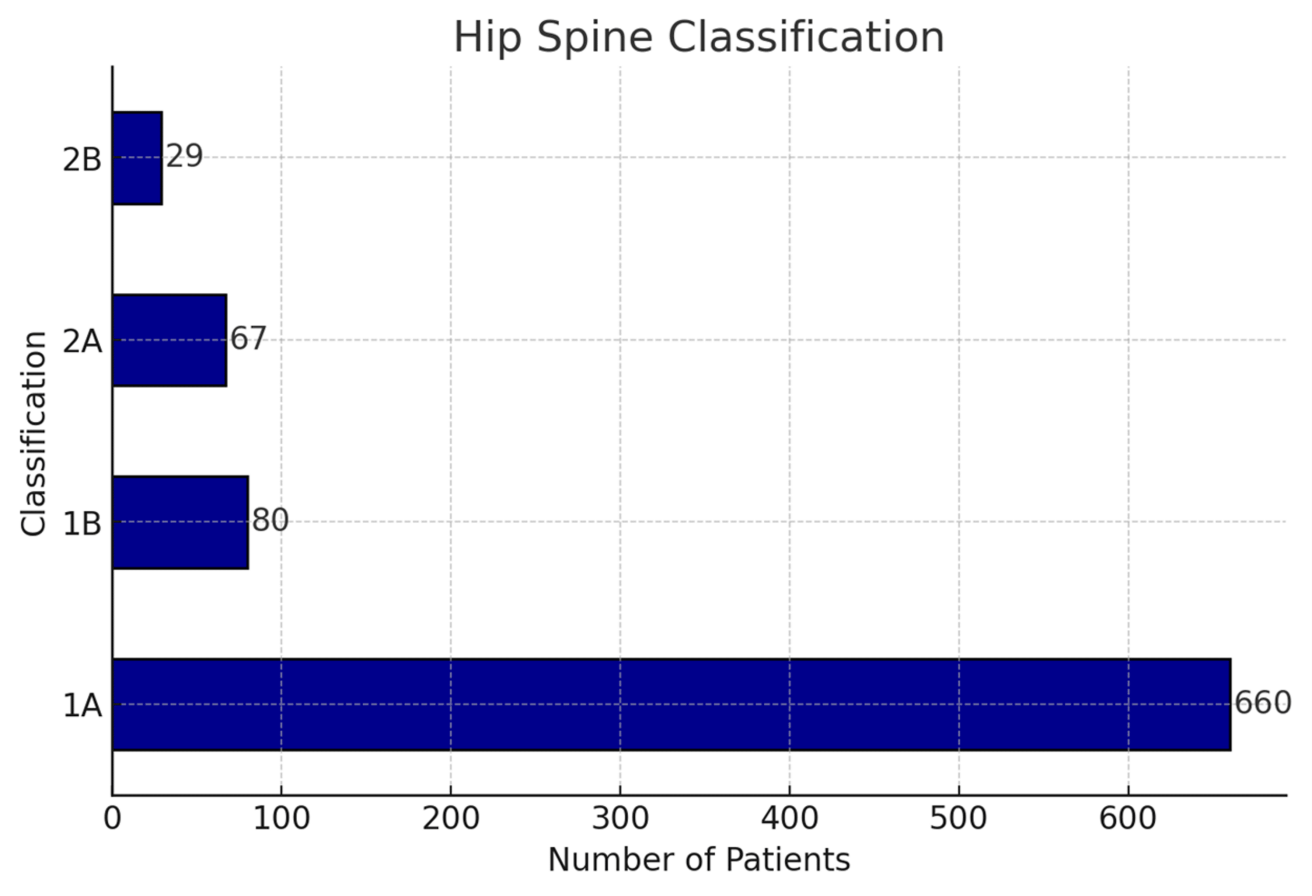
Hip-spine classification in this study.

The breakdown of the patients according to the hip-spine classification was 660 (78.9%) in group 1A, 80 (9.6%) in group 1B, 67 (8%) in group 2A, and 29 (3.5%) in group 2B (Figure 2).

Table 2 summarizes the spino-pelvic parameters by category. The overall mean pelvic incidence, lumbar lordosis, sacral slope (standing), and sacral slope (sitting) in the population were 48.78 (SD=10), 53.88 (SD=9.95), 38.82 (SD=8.85), and 14.61 (SD=11.14) degrees, respectively.

**Table 2:** Category-wise distribution of spino-pelvic parameters in degrees. PI-LL: pelvic incidence-lumbar lordosis.

Classification	Pelvic incidence, mean (SD)	Lumbar lordosis, mean (SD)	PI-LL, mean (SD)	Sacral slope difference, mean (SD)
1A	48.17 (9.17)	55.97 (8.45)	-9.08 (9.49)	27.30 (9.58)
1B	45.46 (10.72)	52.82 (9.35)	-8.27 (10.25)	4.24 (5.66)
2A	58.95 (10.62)	42.89 (9.43)	17.47 (6.00)	25.16 (8.34)
2B	48.36 (11.34)	34.52 (5.59)	20.95 (6.45)	2.29 (5.97)
Overall mean	48.78 (10.00)	53.88 (9.95)	-5.83 (12.75)	24.06 (12.02)

**Figure 3:**
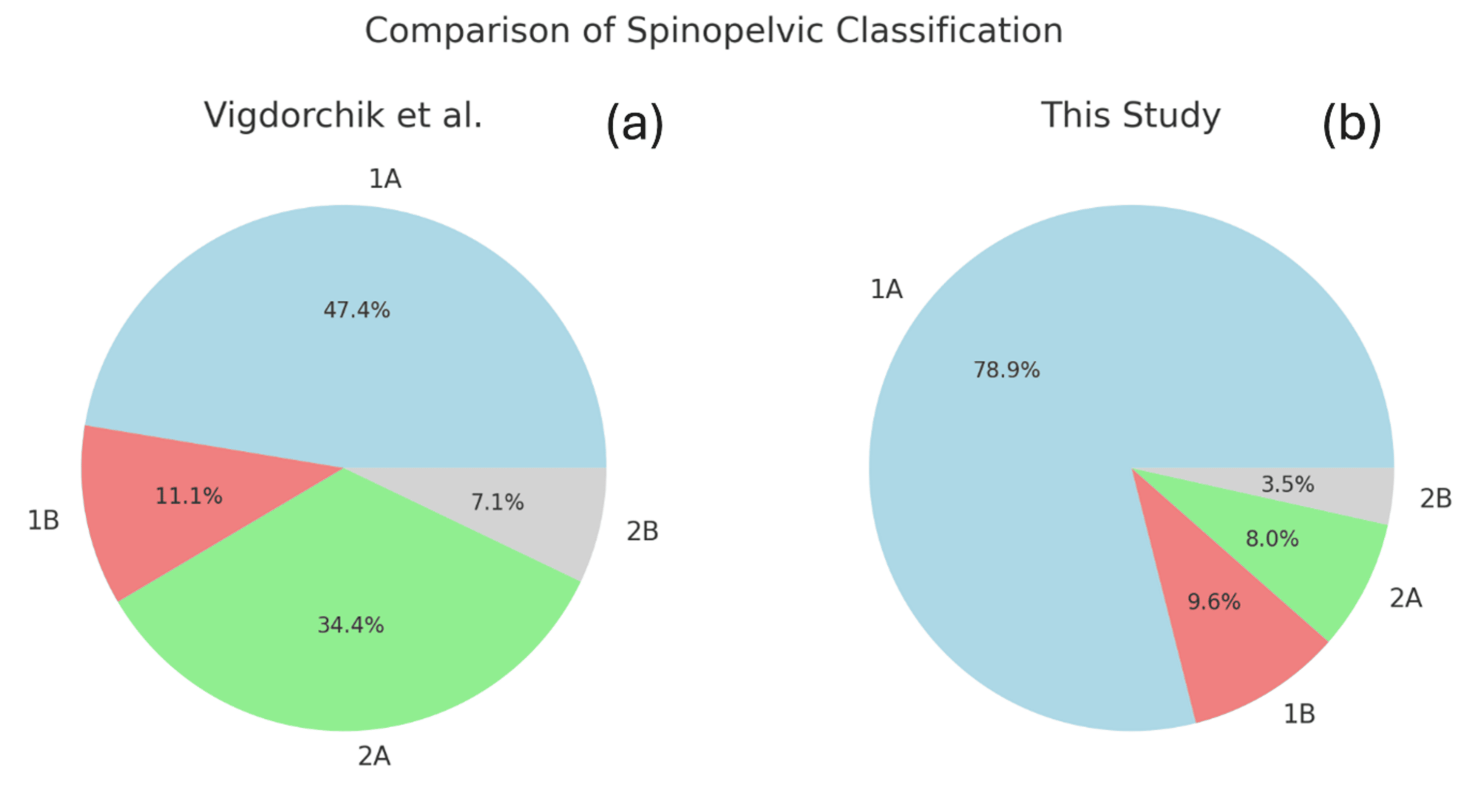
Category-wise hip-spine classification difference between Vigdorchik et al. (a) and the current study (b).

Figure 3 compares our patient cohort to Vigdorchik et al [15]. Our study shows a lesser number of patients under the 2A category, with increased anterior/posterior pelvic tilts due to hip flexion contracture or flatback deformity compensation.

## Discussion

This study aimed to assess the spino-pelvic relationship in Asian patients undergoing total hip arthroplasty (THA) and classify these patients using the hip-spine classification system [15]. The findings provide valuable insights into the spino-pelvic parameters and mobility patterns in this specific patient population, potentially contributing to more tailored surgical approaches and improved outcomes [19]. In this analysis of 836 patients, we observed a predominance of normal spino-pelvic alignment and mobility. The majority of patients (approximately 80%) were classified as type 1A (normal spinal alignment with normal pelvic mobility). The proportion of flatback cases (Group 2A and 2B) was notably lower at 11.5% compared to Vigdorchik et al., in their large multicenter study, which reported approximately 41% of patients having sagittal imbalance (2A or 2B) [15] (Figure 3).

This discrepancy between both studies may be attributed to the insignificant rates of primary hip osteoarthritis (OA) in the Asian population compared to the Caucasian population [20]. The study by Yakkanti et al., which evaluated over 225,000 primary THA patients in the USA database and over 20,000 in the Indian database, showed a significantly lower percentage of primary THA performed for avascular necrosis (AVN) (5.97%) in the US compared to the Indian population (51.8%) [20]. In India, in the absence of an instrumented spine, degenerative spine disease, or a fused hip as seen in cases of ankylosing spondylitis, the majority of young patients undergoing THA fall into Type 1A with a flexible spine [21]. This classification suggests that surgeons may not need to routinely alter acetabular component positioning for these patients. Similar findings were also seen in the study conducted by Łazinski et al., who reported a normal spino-pelvic alignment in the majority 63.1% of their patients [22]. Recognizing that these patients usually maintain normal spinal mobility can simplify preoperative planning and improve surgical outcomes. However, a study by Ohyama et al. showed that only 25.3% of their study population showed normal (1A) spino-pelvic alignment [23]. These findings suggest that the diversity seen in spino-pelvic alignment is multifactorial and that extensive preoperative evaluation is necessary for optimum outcomes after THA [24-26].

A major strength of this study is its pioneering classification of Asian patients undergoing THA based on the hip-spine classification. This provides a foundational understanding of spino-pelvic parameters within this demographic, potentially improving preoperative planning and surgical techniques for Asian patients. Using a standardized classification system facilitates comparisons with international data and enhances the broader understanding of spino-pelvic dynamics in THA. Other studies have reported dislocation rates up to 16% in patients with spine pathology characterized by a PI-LL mismatch of more than 10 degrees [27,28].

Despite the robust sample size, this study has several limitations. First, the retrospective observational design is inherently susceptible to selection bias and unmeasured confounding, as inclusion was dependent on the availability of complete preoperative standing and flexed-seated radiographs. Second, variations in spino-pelvic characteristics may also arise from differences in regions, socioeconomic backgrounds, or healthcare settings, affecting the generalizability of the findings. Additionally, the findings may not be applicable to other ethnic groups or countries, as spino-pelvic parameters can vary due to genetic, cultural, and environmental factors. However, Innmann et al. made an important discovery and showed that even patients with end-stage arthritis of the hip did not exhibit abnormal spino-pelvic parameters when compared to a matched cohort of volunteers [29]. Comparative studies involving diverse ethnic groups and international cohorts are needed to validate the applicability of these results beyond the Asian context. Third, another source of variability arises from the patient's positioning during radiographic imaging. A relaxed-seated position tends to overestimate spinal stiffness by a factor of seven and utilizing a flexed-seated position is recommended for the optimal assessment of spino-pelvic mobility, as implemented in this study [30,31]. Finally, the study did not explore the long-term clinical outcomes and complication rates associated with different spino-pelvic classifications. While the classification provides a framework for understanding spino-pelvic dynamics, its direct impact on post-THA outcomes such as dislocation rates, implant longevity, and patient satisfaction remains to be fully elucidated. Longitudinal studies tracking these outcomes over extended periods are essential to establish the clinical relevance of the spino-pelvic classification system in THA. This is particularly important because a follow-up study of patients who had undergone THA indicated that there was a difference of more than 10 degrees in the standing sacral slope between preoperative and postoperative measurements in 14.2% of patients. The change in the sitting sacral slope was even more significant, with 34% of patients showing differences of more than 10 degrees [32]. A study is currently underway to evaluate changes in the spino-pelvic classification after THA in this cohort of patients and to understand how component positioning affects hip-spine mobility.

Recommendations

Based on our findings, we recommend that surgeons assess spino-pelvic parameters and classify cases, particularly those involving concurrent spine issues or fused hips with stiff spines. Identifying patients with altered spino-pelvic dynamics is crucial for tailoring surgical approaches and optimizing THA outcomes. Incorporating a thorough preoperative evaluation of spino-pelvic alignment will help mitigate the risk of postoperative complications and enhance the overall success rate of THA procedures.

## Conclusions

This study offers important insights into the spino-pelvic parameters of Asian patients undergoing THA and highlights the potential utility of the hip-spine classification system. Assessing long-term clinical outcomes and post-surgical changes in the hip-spine relationship will be crucial for advancing our understanding of spino-pelvic dynamics and optimizing THA outcomes across varied patient groups.
